# Emerging challenges in the transition from manual to automated sleep scoring

**DOI:** 10.1093/sleep/zsaf202

**Published:** 2025-07-21

**Authors:** Thomas Penzel, Matthew Salanitro

**Affiliations:** Sleep Medicine Center, Charité – Universitätsmedizin Berlin, Berlin, Germany; Sleep Medicine Center, Charité – Universitätsmedizin Berlin, Berlin, Germany

Automatic sleep staging in polysomnography (PSG) has reached the accuracy standards defined by the AASM Scoring Manual—a major step forward for sleep medicine. Certified software now performs on par with traditional visual scoring [1], indicating that this once time-consuming aspect of diagnosis may finally be streamlined through automation. Manual scoring—often requiring 1.5 to 2 h per study by an experienced technologist—has long been considered essential, but automation offers the promise of faster, more consistent, and more scalable analysis [2]. This could enable sleep technicians to shift their focus toward tasks such as artifact detection, patient guidance, and quality assurance—ultimately improving both care delivery and data integrity. For high-throughput sleep centers, the potential to reduce workload while maintaining clinical standards is particularly appealing. Automated scoring may also help address long-standing issues such as inter-scorer variability, improving consistency across centers, and enhancing reproducibility in both clinical and research settings [2]. With such benefits on the horizon, automated sleep staging is quickly gaining traction as a core component of the future of sleep diagnostics.

Building on this promising momentum, a recent study sought to evaluate how automated scoring compares to manual scoring in PSGs from patients with suspected obstructive sleep apnea at a single sleep center [3]. As expected, the study found strong inter-scorer agreement between two experienced manual scorers, in line with prior findings [4]. Agreement was assessed using standard statistical approaches, including Bland–Altman plots, and closely matched the ~83% agreement previously reported by Rosenberg. However, a striking result emerged when comparing the manual scorers with the automated software, as the agreement between them was unexpectedly low. This discrepancy is especially notable given the increasing confidence in automated scoring systems, and it highlights that—even as performance improves—automation is not immune to variability. The finding raises important questions about the contextual factors that may influence algorithm performance in real-world clinical datasets. It also serves as a reminder that validation in controlled development settings does not always translate seamlessly to generalizability in clinical practice. As sleep centers continue to adopt automated tools, understanding when and why these systems succeed—or fall short—will be critical. Without such insight, there is a risk of placing too much trust in automation without fully appreciating its current limitations.

Such discrepancies, while potentially concerning, offer valuable insights. They underscore the broader understanding that automation—despite its efficiency and scalability—does not inherently ensure clinical equivalence across all contexts. The divergence observed between manual and automated scoring illustrates that even well-validated algorithms can yield inconsistent results when applied to data that differ from their original development or training environments [5]. This finding invites deeper reflection on the practical challenges of integrating automated scoring into routine clinical practice and the various factors that may affect its reliability. Unlike controlled trials or internal validation pipelines, real-world clinical data are shaped by local scoring protocols, signal variability, equipment differences, and patient heterogeneity—all of which can influence algorithm performance [6, 7]. As such, automation in sleep scoring should not be viewed as a plug-and-play solution but as a dynamic tool requiring ongoing, context-specific evaluation. Ensuring that these systems perform robustly across diverse patient populations, recording conditions, and institutional workflows will be essential for their safe and effective implementation in everyday sleep medicine.

An often-overlooked aspect of algorithm performance is the certification status of the software itself. While many automated scoring systems are well-developed, not all undergo formal evaluation by external bodies such as the AASM. Certification remains a voluntary process, and the absence of it does not necessarily imply that a system is inadequate. However, certification serves as a recognized benchmark for quality and reliability, offering assurance that an algorithm has been tested against established standards [8]. Without this validation, it becomes more difficult to assess whether discrepancies in performance are due to contextual factors, software limitations, or both. As automated tools become more widely adopted, transparency around their development, validation procedures, and regulatory status will be critical to building trust and ensuring consistent clinical application.

That being said, current certification efforts remain limited in scope. The AASM certification framework applies only to sleep stage scoring, leaving other clinically critical domains—such as respiratory event detection—unaddressed [1]. Yet events like apneas and hypopneas play a central role in diagnosis and treatment planning, directly influencing severity classifications and therapeutic decisions. As automated scoring tools begin to tackle these more complex aspects of sleep analysis, the absence of standardized validation protocols becomes increasingly problematic. Expanding certification standards to include respiratory events and other key metrics outlined in the AASM manual would provide much-needed guidance and quality assurance [9]. Until such frameworks are in place, clinicians and researchers must interpret automated respiratory scoring with caution, acknowledging that unvalidated outputs may lead to under- or overestimation of disease burden. Ultimately, broader certification will be essential to ensure the safe, reliable, and clinically meaningful use of automation in sleep medicine.

While certification provides an important benchmark, it does not fully account for how automated systems perform in diverse, real-world settings. Even well-developed algorithms may face challenges when applied across varying clinical environments, where factors such as scoring conventions, technical procedures, and patient populations can introduce variability. Several key sources of such variability deserve closer attention:

(A) **Variation in scoring practice:** Scoring conventions may differ subtly between institutions, regions, or certification bodies. While manual scorers may achieve high agreement within a given center, it remains uncertain whether these patterns align with those used to train automated systems. Such institutional or national “flavors” in scoring practice can introduce unintended discrepancies.
(B) **Technical and procedural differences:** Automated algorithms may be more sensitive than human scorers to minor deviations in signal acquisition—such as electrode placement, sensor configuration, or overall signal quality. Local procedural norms that are clinically acceptable for human interpretation may nonetheless affect automated output.
(C) **Population-specific patterns:** Algorithm performance can also be influenced by demographic and physiological factors. Regional variations in sleep architecture, comorbidities, or medication use may lead to patterns not well-represented in training datasets, especially in retrospective clinical populations. These differences may affect both sleep staging and respiratory event detection in ways that challenge generalizability.

These methodological and contextual factors take on added significance when considering their potential impact on diagnostic classification and downstream treatment decisions. While statistical comparisons—such as differences in AHI distributions or agreement percentages—are useful for benchmarking, they capture only part of the picture [2]. Discrepancies in scoring, whether driven by algorithmic limitations, institutional practices, or population-specific factors, may lead to shifts in diagnosis, treatment thresholds, or follow-up strategies. To fully understand the clinical relevance of automated scoring, future research should move beyond agreement metrics and directly evaluate whether these differences influence real-world decision-making and patient outcomes. Only by linking scoring performance to clinical impact can the field assess the true value—and potential risks—of automation in practice.

To ensure broad applicability, validation of automated scoring systems must extend beyond internal development datasets. Independent evaluations across multiple sleep centers—encompassing diverse equipment, protocols, and patient populations—are critical for assessing generalizability and uncovering potential sources of bias that may remain hidden in more uniform settings. Without this level of external scrutiny, algorithms may misclassify data when deployed outside of their original context, potentially leading to systematic diagnostic or treatment errors.

One promising approach to overcoming the limitations of current automated scoring systems is federated learning (FL), a machine learning technique that enables institutions to collaboratively train models without sharing raw patient data [10]. By keeping data local and exchanging only model updates, FL preserves privacy while allowing algorithms to learn from heterogeneous datasets that reflect variations in protocols, equipment, and patient populations [11]. A recent study demonstrated the feasibility of this approach for automated sleep stage classification using the ODIN platform. In this work, an AI model based on the TinySleepNet architecture was trained across two distinct clinical datasets—one focused on insomnia and the other on obstructive sleep apnea—without centralizing the data [12]. Notably, the model trained in the FL environment performed similarly to one trained using conventional centralized methods, demonstrating that privacy-preserving training can be achieved without compromising accuracy. These findings highlight FL’s potential to support robust, scalable, and privacy-conscious model development in real-world sleep medicine settings. In practice, such an approach could accelerate collaboration across institutions, improve access to diverse training data, and facilitate the deployment of AI tools in clinical workflows without violating data protection laws.

Ultimately, the true value of any scoring method—manual or automated—lies in its ability to support accurate diagnosis and effective patient care. As automated systems continue to evolve, their integration into clinical practice must be guided by independent evaluation, alignment with real-world outcomes, and careful consideration of their clinical implications. Until stronger evidence consistently links automated scoring to diagnostic and therapeutic decisions, these tools should be used as complements, not replacements for expert judgment. Realizing their full potential will require sustained collaboration between clinicians, developers, and regulatory bodies to establish clear standards and use cases. Federated learning, by enabling privacy-preserving collaboration across institutions, may play a key role in supporting this effort. With a measured and evidence-based approach, the field can embrace the advantages of automation while preserving the clinical integrity that underpins high-quality sleep medicine.
